# Influence of the site of acromioplasty on reduction of the critical shoulder angle (CSA) – an anatomical study

**DOI:** 10.1186/s12891-018-2294-1

**Published:** 2018-10-13

**Authors:** Dominik Kaiser, Elias Bachmann, Christian Gerber, Dominik C. Meyer

**Affiliations:** 1 Department of Orthopaedics, University of Zurich, Balgrist University Hospital, Uniklinik Balgrist, Forchstrasse 340, 8008 Zürich, Switzerland; 2 Department of Orthopaedics, Biomechanical Research Laboratory, Balgrist Campus, University of Zürich, Zürich, Switzerland

**Keywords:** Rotator cuff tear, Acromioplasty, Critical shoulder angle, Rotator cuff retear, Digitally reconstructed radiograph, Computed tomography

## Abstract

**Background:**

A large critical shoulder angle (CSA) >35° is associated with the development of rotator cuff tearing. Lateral acromioplasty (AP) has the theoretical potential to prevent rotator cuff tearing and/ or to reduce the risk of re-tears after repair. It is, however unclear which part of the lateral acromion has to be reduced to obtain the desired CSA. It was the purpose of this study to determine which part of the lateral acromion has to be resected to achieve a desired reduction of the CSA in a given individual.

**Methods:**

First, the influence of the exact radiographic projection on the CSA was examined. Second, the influence of anterolateral versus strict lateral AP on the CSA was studied in eight scapulae with different anatomic characteristics. Differences in CSA reduction were investigated using paired t-test or Wilcoxon test.

**Results:**

Scapular rotation in the sagittal and axial plane had a marked influence on the radiologically measured CSA ranging from -6 to +16°. Overall, lateral AP of 5/10mm reduced the CSA significantly greater than anterolateral AP of 5mm/10mm [5mm: 2.3° (range: 0.7°-3.6°) SD±0.8° vs. 1.2° (range: 0°-3.3°) SD±1.1°, *p=0.0002*]/[10mm: 4.8° (range: 2.1°-7°) SD±1.3° vs. 2.7° (range: 0°-5.3°) SD±1.7°, *p=0.0001*]. Depending on scapular anatomy anterolateral AP did not alter CSA at all.

**Conclusions:**

For comparison of pre- and postoperative CSA, the exact orientation of the X-ray and the spatial orientation of the scapula must be as identical as possible. Anterolateral AP may not sufficiently correct CSA in scapulae with great acromial slopes and smaller relative external rotation of the acromion as the critical acromial point (CAP) may be located too posteriorly and thus is not addressed by anterolateral acromioplasty. Consistent reduction of the CSA could be achieved by lateral AP in all eight scapulae.

## Background

The morphology of the scapula shows great differences between individuals. Its variable radiographic appearance has led Bigliani et al. to distinguish three different forms of the acromion as early as 1986 [1]. Later reports focused on different acromial spurs, acromial slope (AS), acromial tilt (AT), lateral acromial angle (LAA), acromion index (AI) and critical shoulder angle (CSA) [2–7]. The interest of these studies is to understand how the scapular anatomy is related to clinical shoulder pathologies and how it might be altered in order to possibly reduce the incidence of degenerative rotator cuff tears or their recurrence after repair [8]. Other reports found a highly relevant impact of a presumably “faulty” body posture on rotator cuff tears, as differences in posture alter the position of the scapula in space [9].

While Moor et al. noted little variation of the radiographic appearance and the CSA in different scapular rotation [10], a newer study has shown a greater susceptibility of the CSA to malposition especially in ante- and retroversion [11].

At our institution all standard AP shoulder radiographs are obtained with the x-ray beam angled 15 degrees caudally in the sagittal plane and vertically in the axial plane, independent of the patient specific scapular morphology especially regarding the acromion, the position in space or the patient’s posture. Using the above-mentioned protocol, it has been shown that a CSA of <28 is highly predictive (odds-ratio >10) for the development of osteoarthritis and a CSA > 35° is highly predictive of rotator cuff tears (RCT) [6]. Consequently, we assume that normalization of a very high (>35°) CSA may be helpful in preventing rotator cuff re-tears and we therefore seek to achieve this goal with acromioplasty in these patients.

**Fig. 1:**
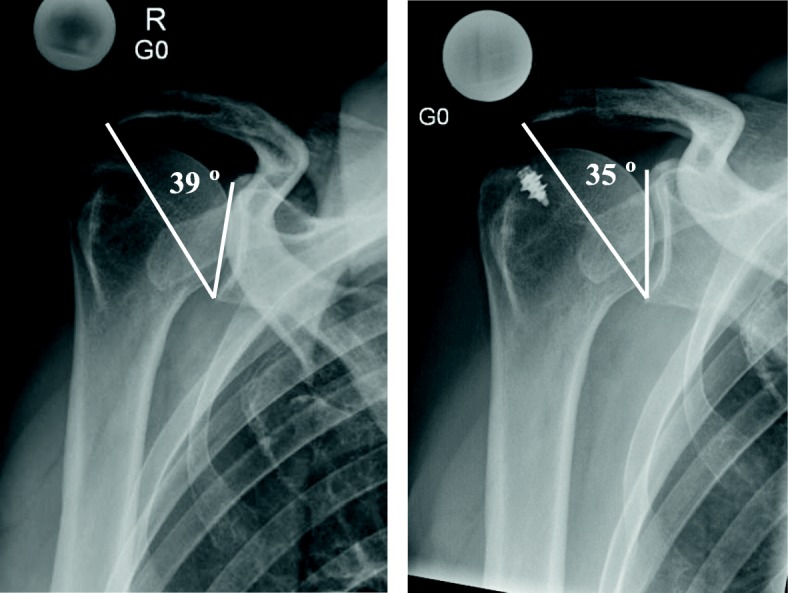
Pre- and postoperative x-ray (*p1 scapula*) reducing CSA from 39° to 35° by anterolateral acromioplasty

We made however the observation that a small resection of an anterolateral acromial spur relevantly decreases the CSA in some patients (Fig. 1), whereas extensive trimming of the whole lateral acromion reduces the CSA only minimally in others. This observation led us to the hypothesis that similar acromioplasties may lead to different corrections of the CSA in different scapular anatomies. Consequently, the first goal of this experimental study was to understand how anterolateral and lateral acromioplasties can have a profoundly different effect on the postoperative radiologically measured CSA as recently described by Katthagen et al. [12]. The second goal was to understand the behavior of the critical acromial point (CAP) in scapulae with different anatomies and in different spatial position [13]. This should help the surgeon achieve a predictable and sufficient correction of a large CSA as defined by Moor et al. [6], while minimizing possible detrimental effects of over- resection of the acromion.

## Methods

### Step 1- Variation of the CSA in three anatomically different scapulae

In a first step, we assessed which point forms the most inferolateral part of the acromion (CAP) on the radiograph. We selected three patients (p1-p3) with distinctly different scapular anatomy regarding acromial slope and relative external rotation of the acromion.

**Fig. 2:**
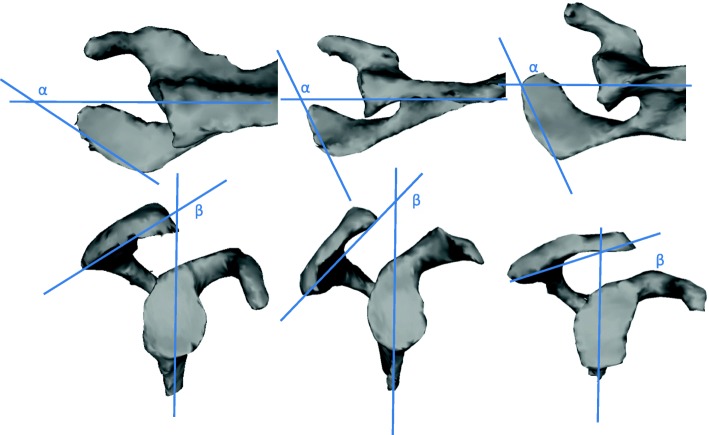
3-D reconstruction of the segmented scapulae showing distinct differences regarding relative external acromial rotation (α – angle between a tangent to the lateral acromial border and a line parallel to the scapular body) and posterior acromial slope (β- angle between a line *connecting the posteroinferior* and the anteroinferior acromion and a line parallel to the scapular body). From left to right *“p1”* scapula, *“p2”* scapula, *“p3*” scapula

All the shoulders of the studied scapulae had a symptomatic rotator cuff tear, operated at our institution and treated with an additional lateral acromioplasty. The MRI Dicom data of these scapulae were segmented using Mimics (Materialise, Leuven, Belgium) and improvement of the models mesh was performed using Meshlab (visual Computing Lab-ISTI-CNR). The segmented scapulae were positioned according to the preoperative true anteroposterior and true lateral view radiographs according to Moor et al. [10] using Blender 2.78 (Amsterdam, Netherlands), a professional open-source 3D computer graphics toolset used for interactive 3D applications. The scapular position was then changed in steps of ±10°, ±20°, ±30°, ±40° flexion/extension and combined with internal/ external rotation up to 10° each. The CSA was measured using Blender 2.78. The relative external rotation of the acromion was defined as the angle between a tangent to the lateral border of the acromion and a line parallel to the scapular body, as seen on an axial radiograph. Posterior acromial slope was defined as the angle of a line connecting the posteroinferior and anteroinferior acromial border and a line parallel to the scapular body as seen on a true lateral view radiograph (Fig. 2), measurements were confirmed by the segmented 3D models.

### Step 2- Effect of lateral vs. anterolateral acromioplasty

**Fig. 3:**
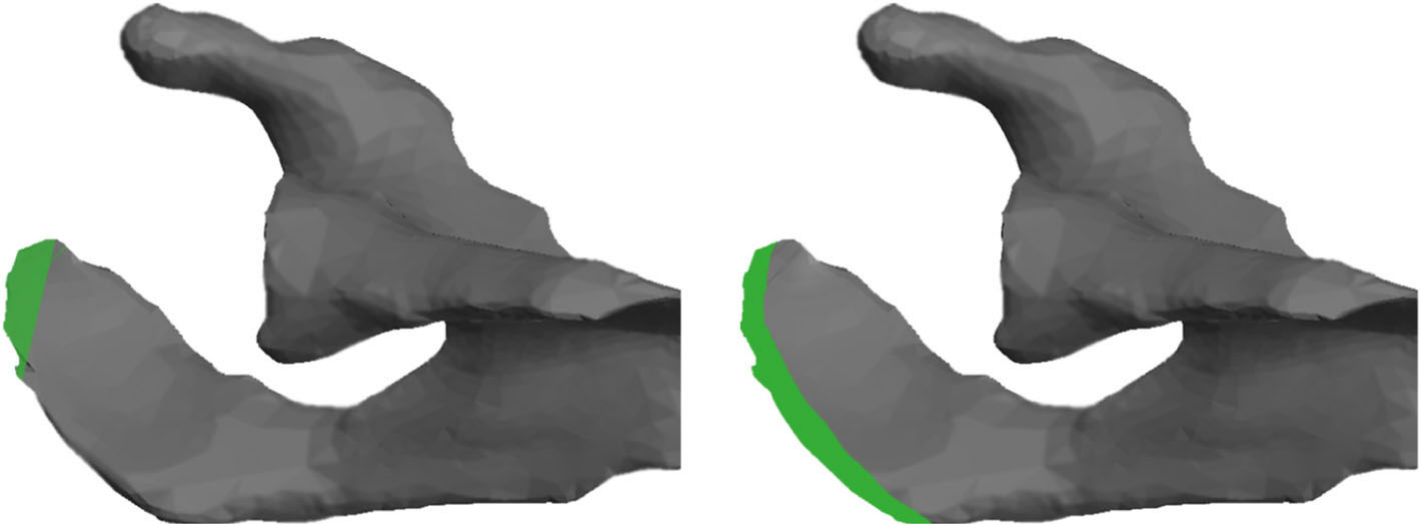
Axial view of the p1 scapula schematically depicting the area of the acromion (green), which is surgically removed during anterolateral (left) and lateral (right) acromioplasty

In a second step lateral and anterolateral acromioplasty of 5mm and 10mm were simulated on the 3 segmented scapulae using Blender 2.78 (Fig. 3). Starting position was defined by the preoperative radiographs as well as the preoperative CSA of the unaltered scapula as measured on a true anteroposterior radiograph according to Moor et al. [10]. Each scapula was then rotated in steps of ±10° from -40° (extension) to +40° (flexion). The CSA was measured in every position as the angle between CAP, inferior glenoid rim and superior glenoid rim.

To increase the validity of the study five additional scapulae (p4-p8) were included. Segmentation of the scapulae from MRI Dicom data, simulation of acromioplasty and CSA measurement were performed identically as described above.

Written informed consent was obtained from all eight patients and Ethic Committee Approval was obtained (KEK Nr.: ZH2016-000826).

### Statistical analysis

Differences in reduction of CSA between anterolateral and lateral acromioplasty in different flexion angles were investigated using paired t-test (or Wilcoxon test, where applicable). *P*-values <0.05 were considered statistically significant. Results are reported with mean, standard deviation and associated p-values if not stated otherwise.

## Results

### Step 1- Variation of the CSA in three anatomically different scapulae

**Diagram 1:**
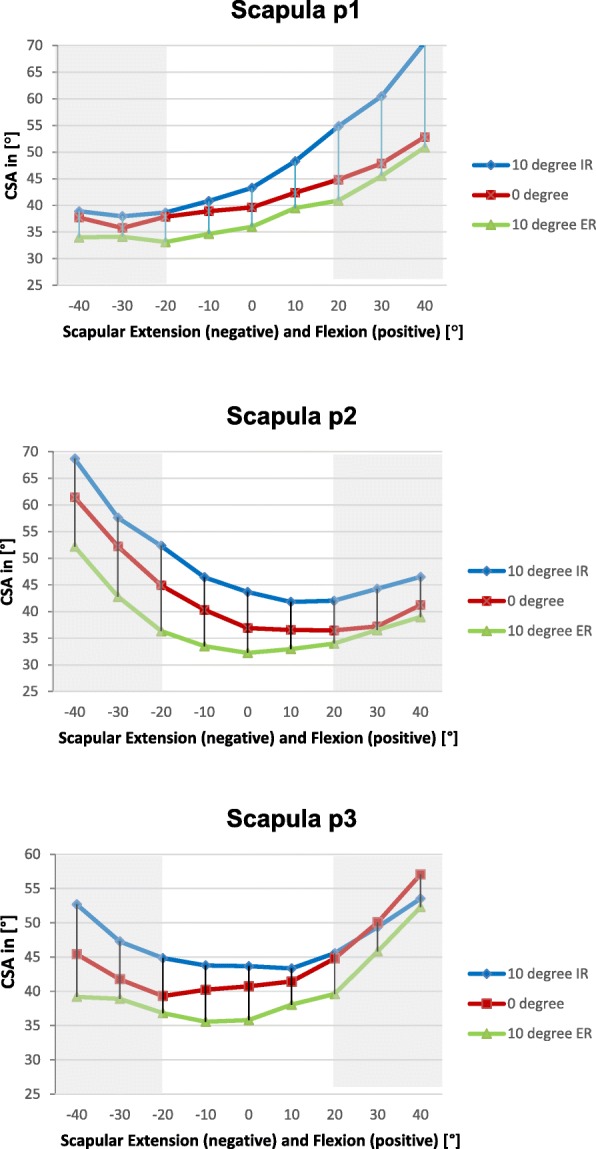
CSA (y-axis) of the three scapulae “p1”, “p2” and “p3” in relation to different extension (negative values) and flexion angles (positive values) (x-axis) in different internal and external rotation, clinically unacceptable rotations highlighted gray

The CSA varied markedly depending on the flexion/extension and internal or external rotation of the scapula as shown in Diagram 1.

Extreme scapular positions, especially ±30° and ±40° flexion/extension, were deliberately included, fully aware that these are unacceptable for clinical use. They were performed to help understand how extreme positions may become relevant for the CSA. In Diagram 1 these are highlighted gray while clinically more likely variations are highlighted white. In clinically likely variations, the CSA varied from 33° to 54° in the “p1” scapula, from 34° to 52° in the “p2” scapula and from 35° to 45° in the “p3” scapula. Internal rotation consistently increases the CSA, while external rotation decreases the CSA in clinically likely variations.

### Step 2- Effect of anterolateral vs. lateral acromioplasty

The first three scapulae were chosen for their distinct anatomical differences and labeled p1, p2 and p3. Five additional scapulae were included to increase validity of the study. These were chosen randomly, segmented and labeled p4-p8. The anatomical characteristics of the eight scapulae regarding preoperative CSA, posterior acromial slope and relative external rotation are summarized in Table 1.

**Table 1:** Anatomical characteristics regarding preoperative CSA, posterior acromial slope and relative external rotation of the 8 examined scapulae (p1-8)

	p1	p2	p3	p4	p5	p6	p7	p8
Preoperative CSA [°]	40	37	40	31	29	37	35	42
Posterior acromial slope [°]	133	145	116	136	117	110	128	102
Relative external rotation [°]	133	114	108	123	132	128	130	111

Overall reduction of the CSA was significantly greater by lateral than by anterolateral acromioplasty of 5mm [2.3° (range: 0.7°-3.6°) SD±0.8° vs. 1.2° (range: 0°-3.3°) SD±1.1°, *p=0.0002*] and significantly greater by lateral than by anterolateral acomioplasty of 10mm [4.8° (range: 2.1°-7°) SD±1.3° vs. 2.7° (range: 0°-5.3°) SD±1.7°, *p=0.0001*].

In neutral position reduction of the CSA did not significantly differ between lateral and anterolateral acromioplasty of 5mm [2.0° (range: 0.7°-2.9°) SD±0.9° vs. 1.1° (range: 0-2.5°) SD±1.1°; *p=0.15*] and between lateral and anterolateral acromioplasty of 10mm [4.4° (range: 2.1°-6.2°) SD±1.5° vs. 2.6° (range: 0-4.5°) SD±1.8°; *p*=*0.06*].

In 10° flexion reduction of the CSA was significantly greater by lateral than by anterolateral acromioplasty of 5mm [2.5° (range: 1.2°-3.3°) SD± 0.7° vs. 1.6° (range: 0°-3.3°) SD± 1.1°; *p*=*0.02*] and significantly greater by lateral than by anterolateral acromioplasty of 10mm [5.3° (range: 3.5°-7°) SD± 1.1° vs. 3.3° (range: 0-5.3°) SD± 1.5°; *p=0.008*].

In 10° extension reduction of the CSA was significantly greater by lateral than by anterolateral acromioplasty of 5mm [2.3° (range: 1°-3.6°) SD± 0.8° vs. 1° (range: 0-2.4°) SD± 1.1°; *p=0.007*] and significantly greater by lateral than by anterolateral acromioplasty of 10mm [4.7° (range: 2.1°-6.3°) SD± 1.2° vs. 2.1° (range: 0-4.6°) SD± 1.7°; *p=0.006*].

**Diagram 2:**
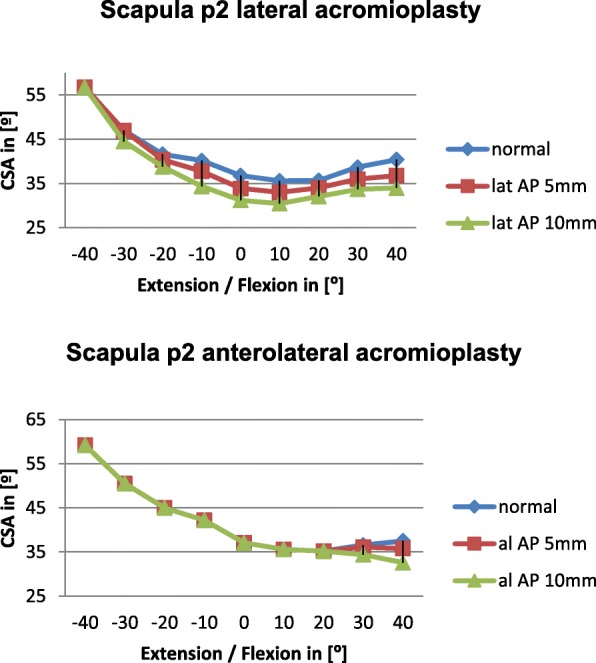
Influence of lateral and anterolateral acromioplasty on the CSA in the p2 scapula dependent on extension and flexion

**Diagram 3:**
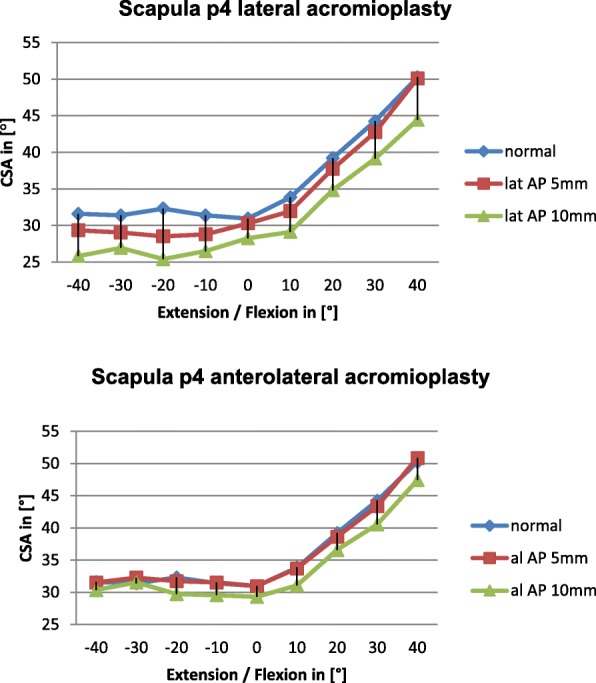
Influence of lateral and anterolateral acromioplasty on the CSA in the p4 scapula dependent on extension and flexion

Anterolateral acromioplasty had no effect on the CSA in 10° extension to 10° flexion in scapulae p2 and a partial effect on p3 (0- 3.3°) and p7 (0-2.8°) (Diagrams 2 and 3).

**Diagram 4:**
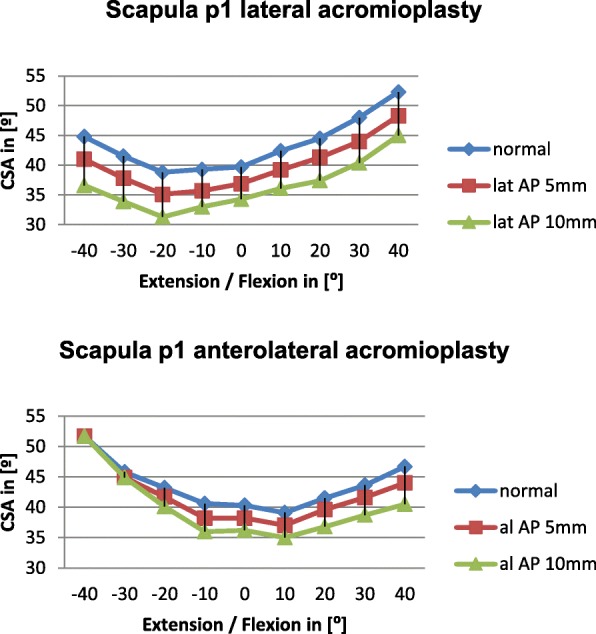
Influence of lateral and anterolateral acromioplasty on the CSA in the p1 scapula dependent on extension and flexion

**Diagram 5:**
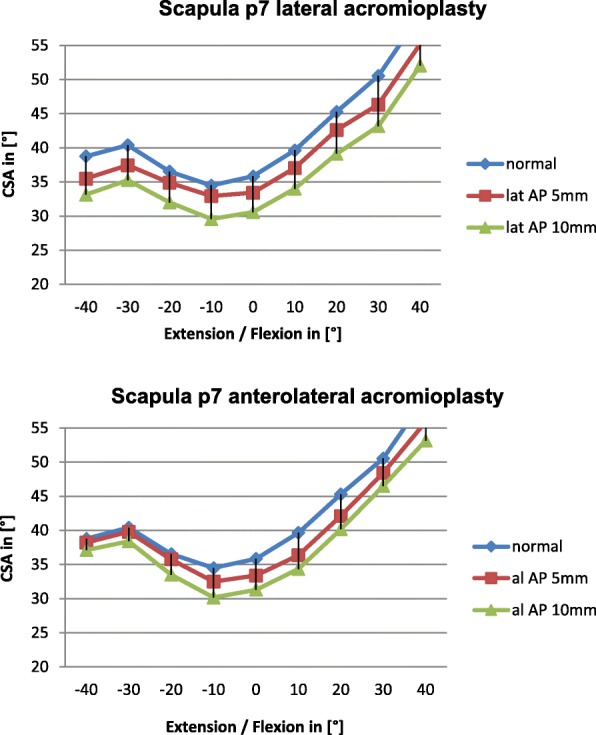
Influence of lateral and anterolateral acromioplasty on the CSA in the p7 scapula dependent on extension and flexion

Anterolateral acromioplasty had a notable effect on the CSA in 10° extension to 10° flexion in the other scapulae with the greatest reduction in scapulae p1 (2.1°-4.6°) and p7 (2°-5.3°). (Diagrams 4 and 5).

The average correction achieved by lateral acromioplasty of 5mm (10mm) was 2.3° (4.7°) in 10° extension, 1.96° (4.44°) in neutral and 2.4° (5.3°) in 10° flexion.

The average correction achieved by anterolateral acromioplasty of 5mm (10mm) increased with increasing flexion of the scapula from 1.2° (2.3°) in 10° extension to 1.3° (2.95°) in neutral and 1.7° (3.70°) in 10° flexion.

## Discussion

Multiple reports [3, 6, 7, 14–16] leave currently little doubt that the radiologically visible lateral extension of the acromion is a relevant predictor for either development of osteoarthritis (“small” acromion) or RCT (“large” acromion). The acromial extension may be measured either by the critical shoulder angle (CSA) or with the acromion index (AI) [7]. Altering these values by acromioplasty during rotator cuff repair may contribute to a lower rate of re-tears, as recently reported by Garcia et al. and Hong et al. [17, 18] even though this has not been widely verified in long-term follow up yet. For rotator cuff repair, the goal at our institution has been arbitrarily set to reduce the CSA to less than 35° in a postoperative follow-up x-ray, as a CSA of greater than 35° has been associated with a higher risk of rotator cuff disease [6, 10].

In our surgical practice however, we made the surprising observation that similar surgical corrections show variable corrections of the CSA. We observed that in one patient (scapula p1) with a preoperative CSA of 39° a purely anterolateral acromioplasty led to a correction of the CSA by 4° to 35° (Fig. 1), while in other patients subjectively similar acromioplasties barely altered the CSA. It was therefore the purpose of this study to understand how lateral and anterolateral acromioplasties can have a profoundly different effect on the postoperatively measured CSA by understanding how the CAP behaves in scapulae with different anatomies and in different spatial position.

**Fig. 4:**
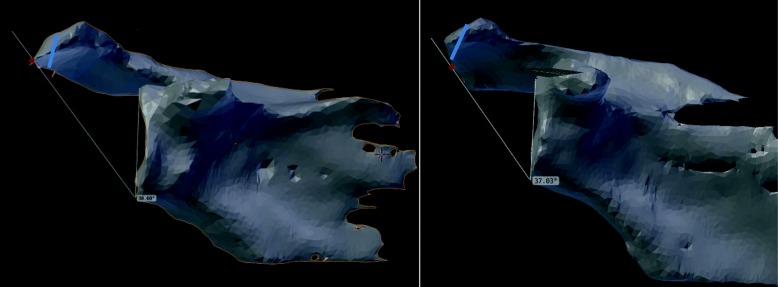
Left: The CAP (red dot) is located anteriorly on the acromion and is affected by the anterolateral acromioplasty. Thus, anterolateral acromioplasty depicted by the blue line does not influence the CSA. Right: The CAP (red dot) is located posteriorly on the acromion and is not affected by anterolateral acromioplaty. Thus, anterolateral acromioplaty depicted by the blue line does not influence the CSA

The analyses of our experimental measurements confirmed our clinical observation in two regards:

1. Great external rotation of the acromion leads to prominence of the anterolateral acromion in defining the CSA. This prominence can be further increased by a bony spur in the AC ligament. The CAP is thus located anteriorly on the acromion and is medialized by strict anterolateral acromioplasty, profoundly reducing the CSA. This applies to our index patient’s scapula where an isolated anterolateral acromioplasty resulted in a similar correction of the CSA as a lateral acromioplasty (Fig. 1, Fig. 4 (left side) and Diagram 4). 2. Anterolateral acromioplasty may lead to little or no change in the postoperative radiological CSA if the CAP is posterior to the site of correction (Fig. 4 (right side)) and therefore a dedicated lateral acromioplasty may be necessary. This seems to occur especially in acromia with greater acromial slope and smaller external rotation.

### Direction of the x-ray beam

Corresponding to the recently published study on the dependency of the CSA of the radiographic viewing perspective of Suter et al. [11] the CSA of the scapulae p1-3 showed a certain positional susceptibility of this clinically valuable parameter even at low angular variations (Diagram 1).

In our second experimental array we did not alter scapular rotation as malrotated x- rays are easily identified at the glenoid and rejected in clinical practice.

### Effect of anterolateral vs lateral acromioplasty

Overall lateral acromioplasty of 5mm and 10mm reduced the CSA significantly greater than anterolateral acromioplasty of 5mm and 10mm, respectively. We believe this is mainly due to the fact, that the CSA is reduced consistently by lateral acromioplasty in all of the included scapulae independent of acromial anatomy. The reduction of the CSA by lateral acromioplasty depends on the amount of bone which is resected (Diagrams 2, 3, 4, 5).

On the other hand anterolateral acromioplasties have a variable effect on the postoperative CSA ranging from no effect at all in scapula p2 (average reduction by lateral AP= 4.1° vs average reduction by anterolateral AP = 0°) to a comparable effect in scapula p1 (average reduction by lateral AP 4.6° vs average reduction by anterolateral AP 3.2°).

We noticed that the CAP seemed to move anteriorly in scapulae with greater external rotation and posteriorly with greater acromial slope. As the CSA is only reduced if the CAP is included in the osseous resection; a more anterior located CAP is more likely to be included by an anterolateral acromioplasty. We concluded that in acromia with greater external rotation and smaller acromial slope, an anterolateral acromioplasty is more likely to reduce CSA.

Further supporting this conclusion is the tendency of greater CSA reduction in increasing flexion of the scapula by anterolateral acromioplasty. Increasing flexion of the scapula relatively reduces the posterior slope, moving the CAP more anteriorly and as mentioned above making it more likely to be included in an anterolateral acromioplasty. Vice versa, increasing scapular extension moves the CAP more posteriorly potentially reducing the effect of anterolateral acromioplasty, which can be seen well in Diagram 4.

There are several limitations to this experimental study. Segmentation was performed by hand using a standardized MRI by the first author. Only eight different scapulae were used to test our hypothesis and simulate acromioplasty. A larger number of test scapulae may be useful to confirm the above mentioned findings, however will most likely not change the intuitively well understandable principal findings we have made. The greatest limitation appears, that we do not know what the real biomechanical effect is that we generate with each acromioplasty procedure.

In summary, a large lateral acromial extension with large CSA and AI may be the result of the projection of a possible anterolateral spur in the coracoacromial ligament especially in scapulae with a smaller posterior acromial slope and a larger relative external acromial rotation (Fig. 1). In such patients, the CAP is located anterior and pure anterolateral acromioplasty may relevantly alter and “correct” the radiologically visible CSA. However, with greater posterior acromial slope and less relative external acromial rotation, the CAP moves further posterior and may exit the area affected by anterolateral acromioplasty. Therefore in those patients, only a lateral acromioplasty will lead to the currently desired reduction of the CSA.

## Conclusion

For comparison of pre- and postoperative CSA, the exact orientation of the X-ray and the spatial orientation of the scapula must be as identical as possible. Anterolateral AP may not sufficiently correct CSA in scapulae with great acromial slopes and smaller relative external rotation of the acromion as the critical acromial point (CAP) may be located too posteriorly and thus is not addressed by anterolateral acromioplasty. Consistent reduction of the CSA was achieved by lateral AP in all eight scapulae.

## Abbreviations

AP: Acromioplasty
CAP: Critical acromial point
RCT: Rotator cuff tears
